# Improving Multiple Pedestrian Tracking in Crowded Scenes with Hierarchical Association

**DOI:** 10.3390/e25020380

**Published:** 2023-02-19

**Authors:** Changcheng Xiao, Zhigang Luo

**Affiliations:** School of Computer Science, National University of Defense Technology, Changsha 410000, China

**Keywords:** multi-pedestrian tracking, hierarchical association, spatial–temporal information, MOT challenge

## Abstract

Recently, advances in detection and re-identification techniques have significantly boosted tracking-by-detection-based multi-pedestrian tracking (MPT) methods and made MPT a great success in most easy scenes. Several very recent works point out that the two-step scheme of first detection and then tracking is problematic and propose using the bounding box regression head of an object detector to realize data association. In this tracking-by-regression paradigm, the regressor directly predicts each pedestrian’s location in the current frame according to its previous position. However, when the scene is crowded and pedestrians are close to each other, the small and partially occluded targets are easily missed. In this paper, we follow this pattern and design a hierarchical association strategy to obtain better performance in crowded scenes. To be specific, at the first association, the regressor is used to estimate the positions of obvious pedestrians. At the second association, we employ a history-aware mask to filter out the already occupied regions implicitly and look carefully at the remaining regions to find out the ignored pedestrians during the first association. We integrate the hierarchical association in a learning framework and directly infer the occluded and small pedestrians in an end-to-end way. We conduct extensive pedestrian tracking experiments on three public pedestrian tracking benchmarks from less crowded to crowded scenes, demonstrating the proposed strategy’s effectiveness in crowded scenes.

## 1. Introduction

Multiple pedestrian tracking (MPT) is a fundamental task which supports many computer vision applications, such as video synopsis, autonomous driving, and intelligent surveillance. The goal of MPT is to generate trajectories of all pedestrians in a video. In the past few years, the tracking-by-detection paradigm [1,2] has dominated this field and achieved great success. This paradigm consists of two separate steps. First, it applies an existing object detector to localize the pedestrians in each video frame with bounding boxes. Second, an association model is designed to link the bounding boxes into complete trajectories using motion or appearance cues. Benefiting from the advance of detection [3,4,5] and re-identification (Re-ID) techniques, the tracking-by-detection-based MPT methods have witnessed great success in easy scenes [6,7,8].

However, such tracking-by-detection MPT methods treat detection and data association as separate steps. As a result, this separate two-step scheme has to face at least two disadvantageous aspects [4,9,10,11]. On the one hand, the biased or false positives easily misguide the tracking process and are hard to rectify. On the other hand, obstacles are posed by the association cues. Many methods [6,12,13] assign detections to tracklets based on appearance similarities for which a separate re-identification neural network is required, making the reference process of tracking complicated. To train the re-identification network, large person re-identification datasets are needed. Moreover, extracting the discriminative features from the heavily occluded pedestrians in crowded scenes is hard.

**Figure 1:**
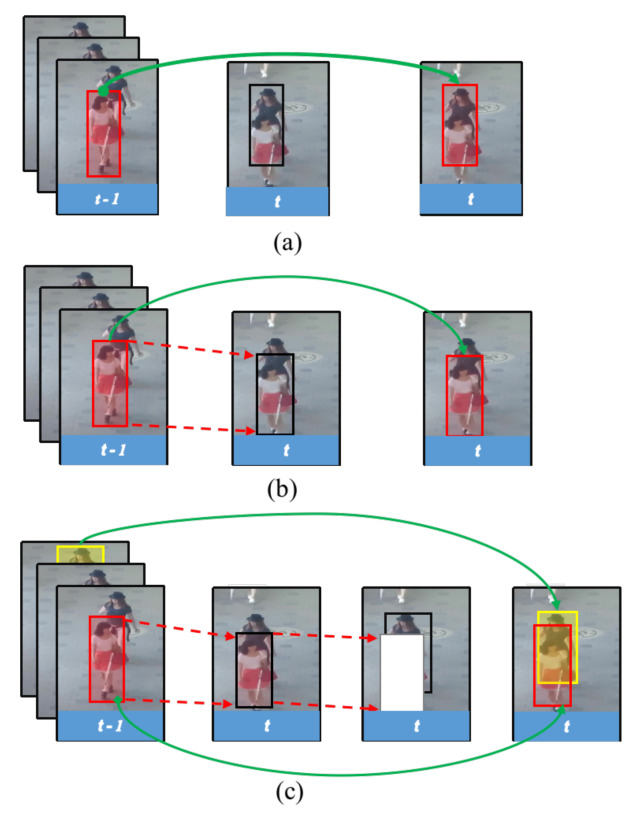
The associations of: (**a**) tracking-by-detection; (**b**) tracking-by-regression; and (**c**) our hierarchical association strategy, for two overlapping pedestrians tracking. In (**a**), the prior detection is directly used to match the same pedestrian; however, the tracking is biased, and another pedestrian is wrongly tracked in the occluded scene. For (**b**), although the regression head directly infers the position of the front pedestrian without the assistance of an additional detector (the red arrow), the back pedestrian is not detected. (**c**) The proposed hierarchical tracking strategy first regresses the front pedestrian (red bounding box), then filters it out implicitly with the learned history-aware mask (white region) to highlight the occluded pedestrian behind. The found pedestrians can further be employed to re-identify inactive tracks (green line) or initialize as a new trajectory for subsequent tracking.

To overcome these defects, we propose a hierarchical association strategy to improve the tracker’s robustness to occlusion and boost the overall tracking performance. Inspired by the idea of Divide and Conquer, we handle targets of varying difficulty hierarchically. The obvious pedestrians are dealt with at the first association and the obscure or partially occluded ones at the second association. At the first association, to make full use of a strong correlation between consecutive video frames, we follow the tracking-by-regression paradigm proposed by Bergmann et al. [11] which exploits the regression head of a two-stage detector, Faster R-CNN [3], to propagate positions of active trajectories from frame *t* − 1 to frame *t*. Then, with the spatial–temporal information provided by the first association, a history-aware mask is constructed to assist the localization of partially occluded pedestrians and small-looking ones. Those refined detections will be assigned to inactive tracks or initialized as new ones. Figure 1 briefly illustrates the difference between the classical tracking-by-detection methods, the tracking-by-regression methods and the proposed hierarchical tracking framework. Moreover, by careful design, our method exploits a simple linear motion model to update positions of inactive trajectories for trajectory rebirth, without the need to train an additional re-identification model to provide appearance information. Our method is simple and achieves competitive performance in many challenging scenes.

We conduct extensive analysis of the proposed tracker on the most widely used multi-pedestrian tracking datasets. The results show the superiority of our approach, especially in severely crowded scenarios.

In summary, the main contributions of our work could be listed as:

- We follow the tracking-by-regression pattern and propose a hierarchical strategy for online multiple pedestrian tracking, especially for crowded scenes. By our deliberate design, the proposed method successfully locates and tracks many small and partially occluded objects.
- We seamlessly incorporate the hierarchical strategy into our tracking framework and capture spatial–temporal cues by constructing a history-aware mask. Thus, we can directly infer both obvious and partially occluded pedestrians.
- Pedestrian tracking experiments on three public multi-pedestrian tracking datasets, from less crowded scenes to very crowded: MOT16 [14], MOT17 [14], and MOT20 [15], show the effectiveness of the proposed method.

This paper is organized as follows: Section 2 presents the review of related works. Section 3 describes the proposed MPT framework with hierarchical association strategy. The effectiveness of the proposed method is validated by experiment results on three standard benchmarks in Section 4. Finally, a summary is provided in Section 5.

## 2. Related Work

### 2.1. Tracking-by-Detection

In the past few years, the tracking-by-detection paradigm has been the prevailing solution to MPT. In this context, the tracking methods can be categorized into offline [16,17,18,19] and online methods [7,20,21,22,23]. Online systems handle video streams to produce trajectories only taking advantage of information up to the current frame. Offline methods, however, can use the whole video sequence as input and process the video frames in a batch. Generally speaking, the online methods have an advantage in time critical-scenes, while offline methods perform better. In this paper, the proposed tracking system follows the online paradigm. SORT [1] uses a linear velocity motion model, namely the Kalman filter [24], to approximate the inter-frame offsets of pedestrians. It then capitalizes on bounding box geometry between neighbor frames to construct an assignment cost matrix and realizes the association by a Hungarian algorithm [25]. Wojke et al. [6] came up with an extension of SORT that integrates appearance information extracted by a pre-trained convolutional neural network (CNN) to improve the tracking performances in scenes with missing detections and occlusion. In order to obtain more robust tracking results, many works [7,8] explore more complex optimization algorithms. Recently, some works have utilized deep learning models to improve data association or to manage the trajectory status [26,27]. Kieritz et al. [28] leveraged a deep multilayer perceptron to guide the tracking process; however, only a fixed number of targets can be processed through time. Furthermore, the deep affinity network (DAN) [2] extracts features of detected objects from selective layers of a VGG-like backbone in a pair of frames and performs exhaustive pairing permutations of features in two consecutive frames to calculate an association matrix. DAN predefines the maximum number of targets that appear in a frame, which cannot work efficiently with the indefinite number of targets among video frames. Liu et al. [29] proposed a graph similarity module to model the relations among pedestrians to acquire more robust affinity information. These mentioned works achieve great success in easy scenes, while few of them explicitly explore tracking issues in crowded scenarios.

### 2.2. New MPT Directions

Several very recent works explore novel MPT paradigms. The joint detection and embedding (JDE) paradigm obtains detections and corresponding appearance representation from a single network. Wang et al. [30] introduced a neural network, which jointly realizes a detection task and an ReID task, yielding detected pedestrians and corresponding ReID features. As successors of this method, FairMOT [10] and CSTrack [31] obtain better performances by balancing the fairness of detection and Re-ID feature extraction. The joint detection and tracking (JDT) paradigm adds a tracking branch to a one-stage object detector to obtain pedestrian motion information between two consecutive frames. CenterTrack [4], a representative work of this kind, takes two continuous frames and detections of a previous frame as input, obtaining detections and trajectories’ offsets for the current frame. TraDeS [32] improves tracking performance of CenterTrack by using tracking cues to assist detection and in return benefit tracking. By sharing most of the calculation between object detection and association cues extraction, these one-shot methods achieve superior performance. Nonetheless, the training of these neural networks needs extra datasets and more carefully refined annotations. Tracktor [11] realizes data association by predicting the corresponding spatial location of tracks in the next frame with the help of a regression head of a detector. Because of this, the tracking-by-regression paradigm needs no track annotations, and it is easy to be transferred to new scenes and has been utilized by some methods [29,33,34,35]. However, this method cannot obtain decent results in challenging scenes. The regression process of active tracks may stagnate when pedestrians occlude each other, and the process needs a separate re-identification model to reactivate. The re-identification feature offered by the Re-ID model may be undiscriminating due to heavy occlusion. Our method follows the tracking-by-regression paradigm; the occluded pedestrians can be associated with corresponding tracklets by the proposed hierarchical association strategy which only uses spatial information.

### 2.3. Tracking in Crowded Scenes

It is hard for object detectors to accurately localize pedestrians when they are not fully visible. Appearance features of pedestrians are often used to associate tracklets and pedestrians, which may fail in crowded scenes due to the undiscriminating features extracted from occluded pedestrians. Gao et al. [36] proposed two models to handle two different types of occlusions, namely, an attention-based appearance model for inter-object occlusion and a scene structure model for obstacle occlusion. TADAM [33] jointly optimizes position estimation and re-identification feature association with mutual benefits, obtaining better performance in heavily occluded scenes. ArTIST [35] utilizes a stochastic autoregressive motion model to both associate tracklets with detections and retrieve inactive tracks. Tokmakov et al. [37] proposed a model which extends CenterTrack [4] with a recurrent memory module. With the help of a synthetic dataset, it can estimate pedestrians’ location when they are fully occluded. Tarasha et al. [38] proposed an online method that forecasts the positions of occluded pedestrians, exploiting depth information from an off-the-shelf monocular depth estimator to handle potential occlusions.

In this paper, we follow the tracking-by-regression paradigm. Different from prior works for partially occluded or small-looking pedestrians, we design a hierarchical association strategy only exploiting spatial information to highlight them in crowded scenes without complex models or additional training data. At the first association, the salient pedestrians are tracked. Then, with assistance from the first association, the partially occluded and small pedestrians are found and assigned to tracklets or initialized as new trajectories at the second association. A simple linear motion model is used to update the positions of inactive tracks for reactivation.

## 3. Proposed Method

**Figure 2:**
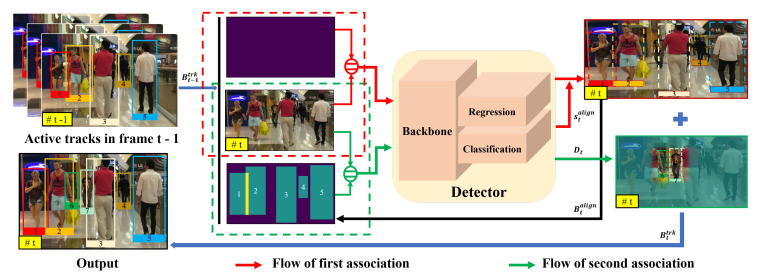
Overview of the proposed hierarchical association strategy for multiple pedestrian tracking. The proposed method consists of two main flows clearly illustrated in red and green, respectively. In the flow of the first association (red), the positions of existing tracks in the *t*-th frame are estimated by the regression head of the detector, according to their previous positions $B_{t-1}^{trk}$ in the frame *t* − 1. In the second flow (green), with the regressed positions $B_{t}^{align}$, a history-aware mask is constructed to assist the detector at this time in highlighting the unmasked regions and further finding the ignored small and occluded pedestrians (the rediscovered small and occluded pedestrian IDs are 6 and 7). The rediscovered targets are used to recover inactive tracks or initialize new tracks. In this way, both obvious pedestrians and obscure ones are assigned to corresponding trajectories (the blue arrow).

We build our model on top of the promising regression-based tracker, Tracktor [11], which propagates spatial locations of active trajectories by the regression head of a two-stage detector. However, this succinct mechanism may fail in challenging scenes, such as motion blur or heavy occlusion. Moreover, a separate re-identification neural network is needed to recover the inactive tracks. We push for progress in tracking pedestrians in these complex scenes by proposing a hierarchical association strategy that follows the divide and conquer idea: The first association for obvious targets and the second for difficult ones, as indicated in Figure 2. Moreover, a simple spatial matching is used to retrieve inactive tracks with the help of a linear motion model instead of a separate re-identification model. It is worth noting that our method does not require any tracking-specific training or sophisticated optimization at inference time, making it easy to transfer to new scenarios where only detection data are available.

### 3.1. Problem Formulation

Given a video sequence $I=\{I_{1},I_{2},\cdots \}$ and corresponding detections $D=\{D_{1},D_{2},\cdots \}$, where provided detections of frame *j* is $D_{j}=\left\{b_{j}^{1},b_{j}^{2},\cdots \right\}$, the task of multiple pedestrian tracking is to produce a trajectory set $T=\{T_{1},T_{2},\cdots \}$. We denote a trajectory $T_{i}$ as a list of ordered bounding boxes $T_{i}=\left\{b_{t_{1}}^{i},b_{t_{2}}^{i},\cdots \right\}$; each pedestrian $b_{t}^{i}=\left(x,y,w,h\right)$ is described by the top left corner image coordinates, width and height, and *t* is the timestamp of the video frame.

### 3.2. Network Architecture

**Figure 3:**
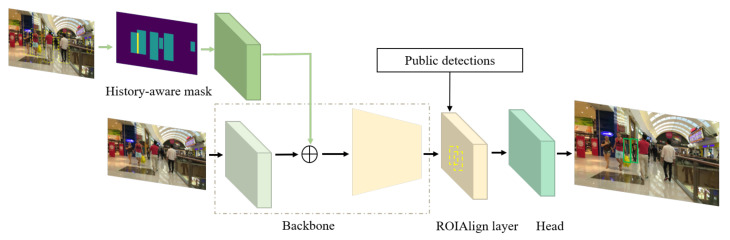
Network architecture of the second association. The network takes a video frame and history-aware mask as input, and the public detections as proposals for the RoIAlign layer. The fusing block is marked in deep green.

We build our method upon a two-stage object detector, faster R-CNN, which consists of two major components, a region proposal network (RPN) and a region-based detection network. Faster R-CNN takes a video frame $I_{t}\in R^{3\times H\times W}$ as input and produces feature map $f_{t}=B\left(I_{t}\right)$ by the backbone network B(·). We build our tracker by adding an extra input branch on the backbone of faster R-CNN, a history-aware fusing block, which takes a history-aware mask $H_{t}\in R^{1\times H\times W}$ as input, as shown in Figure 3.

The resolution of the history-aware mask is the same as the input image. To maintain consistency, we build the history-aware mask for both stages of hierarchical association; even the history-aware mask does not provide any information at the first association. The build process is as follows: in the first stage of data association, the value of every pixel in the history-aware mask is set to 0. In the second stage of data association, we construct the history-aware mask based on the aligned boxes of active tracks in frame $I_{t}$, which are derived from the first stage of data association. Suppose the aligned boxes set of active trajectories in frame $I_{t}$ is $B_{t}^{align}={\left(x_{k},y_{k},w_{k},h_{k}\right)}_{k=1}^{n}$. The history-aware mask for the second stage is constructed as follows:

(1) $$H_{t}=\sum_{k=1}^{|B_{t}^{align}|}1,x_{k}\leq x\leq x_{k}+w_{k},y_{k}\leq y\leq y_{k}+h_{k}.$$

Therefore, the value of each pixel for the history-aware mask equals the number of aligned boxes that cover the pixel. The history-aware mask is processed by the fusing block, a convolution layer. Then, the extracted history feature map is added with the activation of the first convolution layer of the backbone of faster R-CNN. In our case, the convolution layer that processes the history mask has 64 filters with kernel size 3 and stride 2. In order to enable the network with the ability to detect difficult pedestrians in challenging scenes, we train the model only with detection annotations in an end-to-end way. During the training process, inspired by IterDet [39], we randomly split the ground truth detection bounding box set $B_{gt}$ of frame *t* into two subsets $B_{his}$ and $B_{redis}$ with $B_{his}\cup B_{redis}=B_{gt}$ and $B_{his}\cap B_{redis}=\emptyset$. We consider the aligned boxes set as $B_{his}$ in the first stage of data association and employ it to construct the history-aware mask $H_{t}$. We regard $B_{redis}$ as samples to force the network to discover difficult pedestrians that are missed in the first stage. Consequently, a well-trained network has the ability to find out the missing pedestrians given already found pedestrians. Moreover, this training method provides an additional source of data augmentation by different splits of $B_{gt}$. The loss function for the network is defined as follows:

(2) $$L\left(c,\hat{c},b,\hat{b}\right)=L_{cls}\left(c,\hat{c}\right)+L_{reg}\left(b,\hat{b}\right).$$

where $\hat{c}$ and $\hat{b}=\left\{x,y,w,h\right\}$ are the predicted confidence score and bounding box geometry, and *c* and *b* are the corresponding labels. The learning objective of training consists of two loss functions, namely, the object classification loss $L_{cls}$, and the bounding box regression loss $L_{reg}$. The classification loss $L_{cls}$ is formulated as a cross-entropy loss and the regression loss $L_{reg}$ as a smooth L1 loss.

### 3.3. Inference Algorithm

In the beginning, our method initializes tracks $B_{0}^{trk}=B_{0}$ using the set of public detections at frame t=0. The overview of the hierarchical data association strategy is shown in Figure 2.

#### 3.3.1. First Association

As indicated with red arrows in Figure 2, the first stage data association for active trajectories is achieved by the regression head of the network. Specifically, the network takes current frame $I_{t}$, the bounding boxes $B_{t-1}^{trk}$ of active tracks in frame $I_{t-1}$ and the blank history-aware mask as input, among which $B_{t-1}^{trk}$ are the proposals to the RoIAlign layer [40], the regression head returns the potential locations $B_{t}^{align}$ and corresponding confidence scores $s_{t}^{align}$ in current frame *t*.

Consequently, the identity index $\left\{k_{1},k_{2},\cdots ,k_{n}|\;\leq k_{1},k_{2},\cdots ,k_{n}\leq N\right\}$ of active tracks is inherited from frame t−1. If confidence scores in $s_{t}^{align}$ are lower than a threshold $\delta_{active}$, it indicates that the corresponding tracks are potentially disappeared or occluded and should be set inactive.

#### 3.3.2. Second Association

The emergence of new trajectories and the re-emergence of inactive ones occur gradually, during which the pedestrians are partially occluded and and some appear small. It is difficult to extract useful semantic information from these occluded and small targets, making them easily overlooked at the first association. In order to improve the tracking robustness to these targets, we exploit the second data association, as indicated by the green arrows in Figure 2. At the second stage, we construct the history-aware mask according to the aligned bounding boxes $B_{t}^{align}$ of active tracks. At this time, the network takes the current image $I_{t}$ and the constructed history-aware mask $H_{t}$ as input. To make a fair comparison with other tracking methods in the widely recognized datasets, we also use the public detections as the proposals to the RoIAlign pooling layer as shown in Figure 3. By our setting, the head of the network returns the overlooked targets $D_{t}$ as indicated in Figure 2 with green arrows. They are preferentially associated with the trajectories which turned inactive at first association based on spatial similarity. Then, the remaining pedestrians are assigned to tracks set as inactive previous to the current frame or initialised as new tracks. Only when the confidence score of a detection from $D_{t}$ is larger than a threshold $\gamma_{new}$, will the detection be assigned to an inactive trajectory or initialize as a new one. To associate new detections with inactive trajectories, instead of using a re-identification model to acquire appearance cues for the association, we use a linear motion model (LMM) to update their positions for spatial matching based on IoU.

The whole tracking process is shown in Algorithm 1. Like many previous works [1,6,10,11,30], a trajectory is abandoned if it is not assigned with new detections for consecutive *N* frames.
**Algorithm 1** The proposed tracker.**Input:** Video sequence $I={\left\{I_{t}\right\}}_{t=0}^{N-1}$ of frame $I_{t}$ at time *t* and public detection set $D={\left\{D_{t}\right\}}_{t=0}^{N-1}$ of detections $D_{t}$ for frame $I_{t}$.**Output:** Trajectory set $T={\left\{T_{k}\right\}}_{k=0}^{M}$, with $T_{k}=\{b_{t_{1}}^{k},b_{t_{2}}^{k},\cdots ,b_{t_{M}}^{k}\left|0\leq t_{1},\cdots ,t_{M}\leq N-1\right\}$ as a list of ordered object bounding boxes $b_{t_{i}}^{k}=\left(x,y,w,h\right)$.1:$T,T_{active}\leftarrow \emptyset$2:**for**$I_{t},D_{t}$ in zip(I,D) **do**3:    **if** t==0 **then**4:        Initialize the active trajectory set $T_{active}$ with $D_{t}$;5:    **else**6:        // Estimate new positions of inactive tracks7:        T← LMM(T)8:        $B^{align},S^{align},T_{active\_remain}\leftarrow \emptyset$9:        $H_{mask}\leftarrow$ Mask_construction(*∅*)10:        **F** ← Backbone($I_{t}$, $H_{mask}$)11:        // First association12:        **for** $T_{k}\in T_{active}$ **do**13:           $b_{t-1}^{k}\leftarrow T_{k}\left[-1\right]$14:           $b_{t}^{k},s_{t}^{k}\leftarrow$ Detector.RoI_head(**F**, $b_{t-1}^{k}$)15:           **if** $s_{t}^{k}<\delta_{active}$ **then**16:               $T_{active}\leftarrow T_{active}\setminus T_{k}$17:               $T_{active\_remain}\leftarrow T_{active\_remain}\cup \left\{T_{k}\right\}$18:           **else**19:               $B^{align}\leftarrow B^{align}\cup \left\{b_{t}^{k}\right\}$20:               $S^{align}\leftarrow S^{align}\cup \left\{s_{t}^{k}\right\}$21:           **end if**22:        **end for**23:        $H_{mask}\leftarrow$ Mask_construction($B^{align}$)24:        **F** ← Backbone($I_{t}$, $H_{mask}$)25:        $B_{t},S_{t}\leftarrow$ Detector.RoI_head(**F**, $D_{t}$)26:        // Second association27:        Associate $T_{active\_remain}$ and $B_{t}$ using IoU distance28:        $T_{active\_re\_remain}\leftarrow$ remaining tracks from $T_{active\_remain}$29:        $B_{t\_remain},S_{t\_remain}\leftarrow$ remaining detections from $B_{t},S_{t\_remain}$30:        $T\leftarrow T\cup T_{active\_re\_remain}$31:        Associate **T** and $B_{t\_remain}$ using IoU distance32:        $B_{t\_rest},S_{t\_rest}\leftarrow$ remaining detections from $B_{t\_remain},S_{t\_remain}$33:        **for** $d_{t},s_{t}$ in zip($B_{t\_rest},S_{t\_rest})$ **do**34:           **if** $s_{t}>\gamma_{new}$ **then**35:               $T_{j}\leftarrow \emptyset$36:               $T_{j}\leftarrow T_{j}\cup \left\{d_{t}\right\}$37:               $T_{active}\leftarrow T_{active}\cup \left\{T_{j}\right\}$38:           **end if**39:        **end for**40:    **end if**41:**end for**42:$T\leftarrow T\cup T_{active}$

## 4. Experiments

In this section, we test the tracking performance of the proposed method on the commonly used datasets in the MOT field. Comparing our method with the latest published MOT approaches indicates that our method establishes a new state of the art, especially in complex scenes where occluded pedestrians and small looking ones occur frequently.

### 4.1. Datasets and Evaluation Metrics

We conduct experiments on the MOTChallenge benchmarks (https://motchallenge.net/, accessed on 20 December 2022), including MOT16, MOT17 and MOT20. The video sequences in these datasets are taken by static or moving cameras in real scenes under various weather conditions, viewpoints and illumination. The MOT16 benchmark contains 7 annotated training videos and 7 testing videos with DPM detection results provided. The MOT17 includes the same sequences as MOT16 with more accurate annotations. The public detection of MOT17 are obtained by three object detectors with increasing performance, DPM [41], faster R-CNN [3], and SDP [42]. The MOT20 benchmark is the latest released datasets, consisting of eight video sequences taken in very crowded scenes, among which four sequences are for training and four sequences for testing. The MOT20 provides faster R-CNN [3] detection results.

In order to evaluate the performance of our proposed approach quantitatively, we adopt the CLEAR MOT (multiple object tracking) metrics [43], i.e., the multiple object tracking accuracy (MOTA) and the multiple object tracking precision (MOTP) that fuse three sources of errors: false positives (FP), false negatives (FN) and the identity switches (IDS). The IDF1 Score metric is utilized to quantify the identity preservation ability.

We perform all experiments with the public detections provided by MOTChallenge to make a fair comparison with other advanced tracking approaches. Just like previous works, Tracktor [11] and TADAM [33], our method initializes a new trajectory only with a public detection bounding box, and we consider our method as public.

### 4.2. Implementation Details

#### 4.2.1. Training

We employ ResNet50 [44] with a feature pyramid network (FPN) [45] pretrained on ImageNet [46] as the backbone of the proposed network. We train two separate models for MOT16/MOT17 and MOT20 following previous works [4,10,11,30] since there are significant gaps between them. We train the proposed model only with detection annotations.

In the training process, we use stochastic gradient descent (SGD) with *momentum* = 0.9 and *weight_decay_* = *e*^−4^ as optimizer. We train our detector for 12 epochs on a single RTX 2080Ti GPU on MOT17 with provided faster R-CNN detections and 24 epochs on MOT20, with a batch size of 2. Moreover, the learning rate is initialized to 0.02 and divided by 10 after the 8th and 11th epochs for training on MOT17 and divided by 10 after the 16th and 22nd epochs for training on MOT20.

#### 4.2.2. Inference

As stated earlier, the inference of our method is determined by two parameters: The confidence score threshold *δ_active_* at the first association and the confidence score threshold *γ_new_* at the second association. We empirically set *δ_active_* = 0.5 and *γ_new_* = 0.5 to perform experiments on all benchmarks.

### 4.3. Benchmark Evaluation

We evaluate our method on the test sets of MOT16, MOT17 and MOT20 with the public detections provided by the official MOTChallenge and compared with other advanced methods. To better demonstrate the performance of our model, we list both offline and online trackers. For a fair comparison, our method is only compared with online methods published with peer reviews.

As shown by the results in Table 1, Table 2 and Table 3, our method attains state-of-the-art results on three widely used benchmarks without complex post-processing or optimization. Thanks to the proposed hierarchical association strategy and history-aware fusing block, our method has the ability to track the partially occluded pedestrians and the small-looking ones, proved by the excellent false negative results. By alleviating the tracking problem of hard pedestrians, performance improves in many aspects. On the one hand, our methods yield excellent performance in false negatives. This means that our tracker tracks as many pedestrians as possible, usually those that are partially obscured or appear smaller; on the other hand, generally, there exist more FN than FP and IDs. With the significant reduction of FN, the MOTA metric (MOTA is defined as $1-\sum_{t}\left(FN_{t}+FP_{t}+IDs_{t}\right)/\sum_{t}GT_{t}$) is improved directly. As a result, our method outperforms other competing trackers by a noteworthy margin for the metric of MOTA.

Table 3 contains the test results on the most challenging MOT20 benchmark, which includes video frame sequences from known and unknown scenes, in which the mean pedestrian density reaches 246 per frame, which is 10 times higher than in MOT16 as well as MOT17. It is seen that the proposed method achieves the best performance among the online methods, including the previous work Tracktor++v2 (+7.3 MOTA) and state-of-the-art TADAM [33] (+3.3 MOTA). Our method outperforms the second best tracker by 3.7 IDF1 on MOT20. It is worth noting that even though our approach does not exploit a separate re-identification neural network to assist data association, our method obtains the best IDF1, proving the superiority of our method in maintaining the identity of pedestrians.

**Figure 4:**
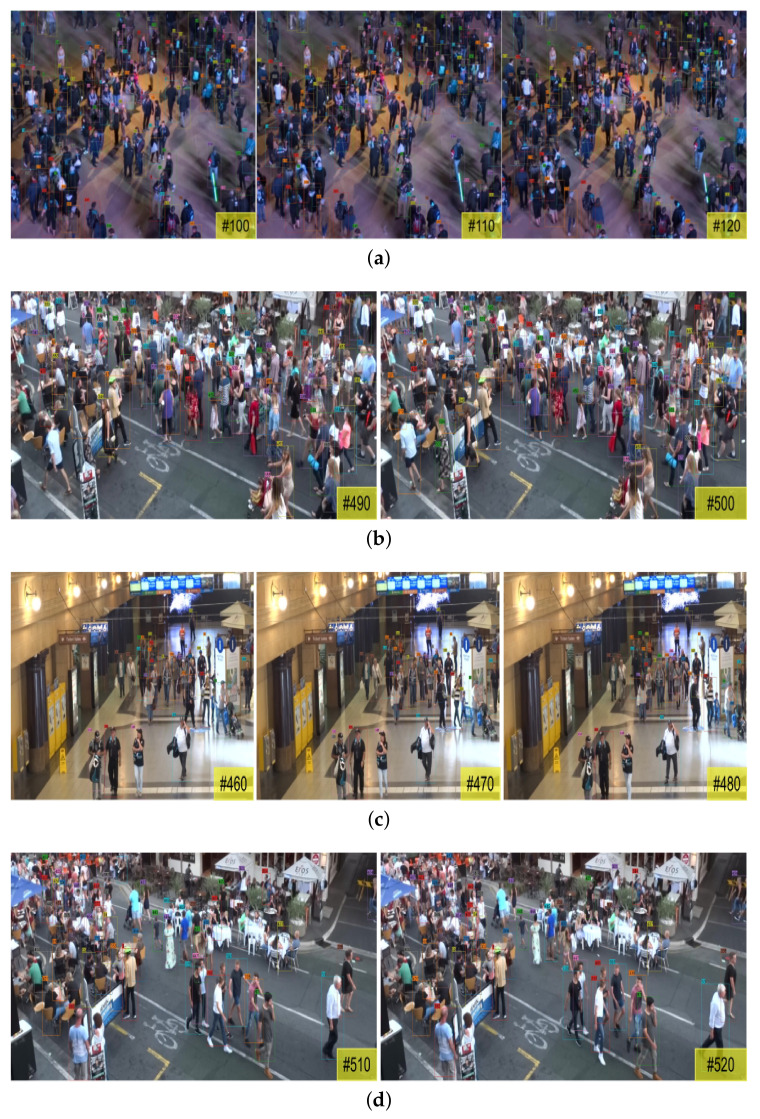
Qualitative results on all four test sequences of the MOT20 benchmark. The datasets are captured in very crowded scenarios in which pedestrians seriously occlude each other with severe variation of illumination. Especially, the MOT20-06 and MOT20-08 are captured in the newly introduced scene that is not included in the training data: (**a**) MOT20-04; (**b**) MOT20-06; (**c**) MOT20-07; (**d**) MOT20-08.

Qualitative results of our method on the MOT20 test set are illustrated in Figure 4. The bounding box colors and the unique identity number on the top left of the bounding boxes indicate the obtained trajectories. It can be clearly found in the figure that our tracker realizes a decent performance in the extremely crowded scenes. The superior results on the unknown scene, MOT20-06 and MOT20-08, indicate that our model has good generalization capability.

### 4.4. Ablation Studies

In this section, to make a deep quantitative analysis of the proposed algorithm. We conduct ablative experiments on the very crowded datasets: MOT17 and MOT20. Because the dataset only provides a training set and a test set, we split each video sequence in training set in half, the first half for training and the last for validation.

**Effectiveness of the hierarchical association strategy**. In this section, we explore the effectiveness of the proposed hierarchical association strategy. We use the previous work, Tracktor [11], as a contrast in Table 4. The *w*/*o His* in Table 4 signifies the implementation of the proposed tracker without using spatial–temporal information. To use it our experiment, we set the pixel values of the history-aware mask to be zeros in both the first and second association. Without the information of active trajectories, the obvious pedestrians will be detected; just like Tracktor, we add an NMS step to suppress detections which overlap active tracks. The *w*/*o His* obtains a 0.6 percentage point improvement compared with Tracktor from 70.6% MOTA to 71.2% MOTA. At the second association, the tracker uses the public detections as proposals for the RoIAlign layer, and with the help of the history-aware mask, the partially occluded pedestrians and the small-looking ones will not be missed. The history-aware fusing block in the proposed model implicitly excludes the already tracked pedestrians in the first association, and thus makes our tracker focus on the remaining difficult pedestrians. To confirm the effectiveness of the history-aware fusing block in finding difficult pedestrians in the second association, we construct a history-aware mask as a definition by Equation (1) and observe that the full model realizes a further 1.3 percentage point improvement from 71.2% to 72.5% compared with *w*/*o His*.

**Figure 5:**
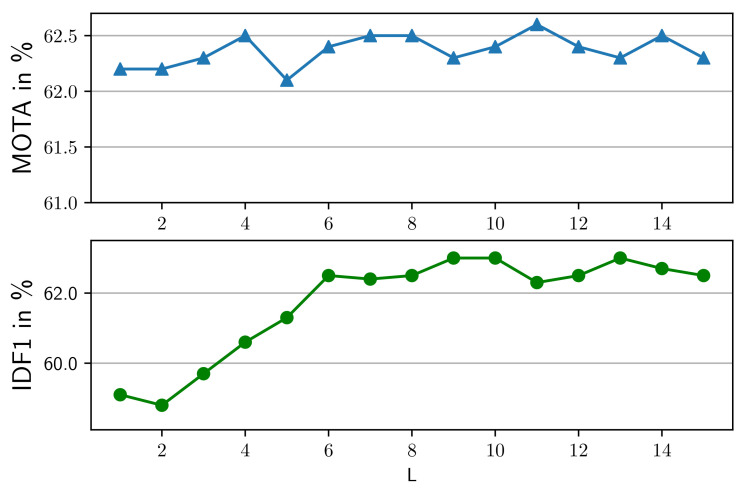
Different number of previous frames: We show the effect of using different numbers *L* of previous frames in a linear motion model on tracking performance.

**Effects of motion models**. We conducted an ablation study of the motion model in our method on the MOT17 validation dataset, which contains video sequences with different motion information captured by surveillance cameras, in-vehicle cameras and handheld cameras. At first, a camera motion compensation (CMC) model is exploited to align trajectories with the current frame. Then, before the second association, a linear motion model (LMM) is applied to update positions of inactive trajectories where we assume pedestrians move in a constant velocity. As shown in Table 5, both CMC and LMM improve the overall tracking performance. This combination achieved the best overall tracking performance, with a 4.3 and 12.3 percentage point improvement in MOTA and IDF1, respectively, compared to the case without the motion models. Therefore, we employ both CMC and LMM. We keep the parameter settings of CMC in Tracktor [11]. To estimate the position of an inactive track in the frame *t*, we calculate its velocity by averaging the offsets of bounding box centers in the last *L* frames. As indicated in Figure 5, at *L* = 9, our method yields the best IDF1. With the increase of *L*, MOTA is better. The estimated velocity is more accurate with more bounding boxes of a track considered. Considering MOTA and IDF1 together, we set *L* = 9.

**Figure 6:**
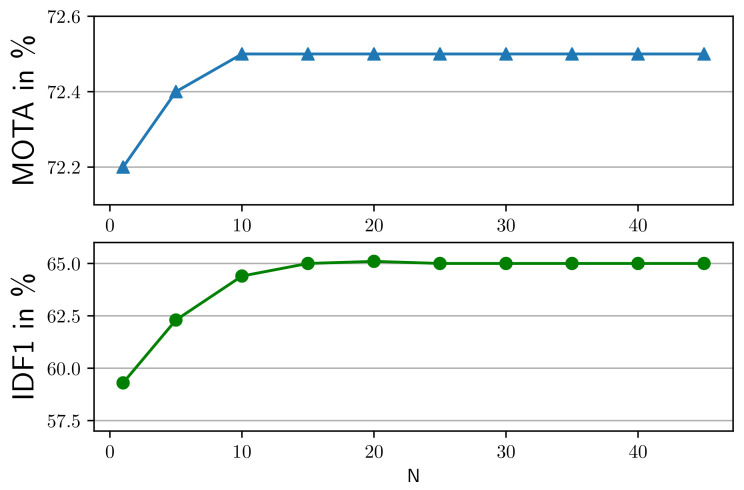
Effect of parameter N. If a track is set to inactive, it retains only consecutive N-frames.

**Influence of number of frames retaining inactive tracks**. In this subsection, we study the impact of the number of frames retaining inactive tracks on performance. The proposed method focuses on improving local tracking performance just like Tracktor [11]. However, after pedestrians occlude each other, a pedestrian can be visible again. This is common in crowded scenes in MOT20. To combat this, we keep a trajectory for *N* frames until it fails to associate with a corresponding target. As illustrated in Figure 6, the tracking performance increases with *N*, especially IDF1 that focuses on the temporal consistency of trajectories. This indicates our tracker can track pedestrians beyond a few consecutive frames. The improvement stops around *N* = 20; we set *N* = 20. In future work, we will explore improving our tracker to track through longer occlusions.

### 4.5. Analysis

**Figure 7:**
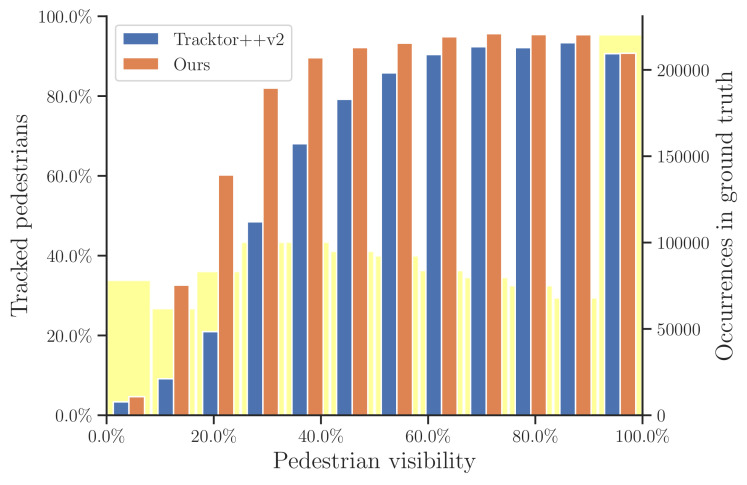
Illustration of the ratio of tracked pedestrians with respect to visibility on the MOT20 training set. The transparent yellow bars indicate the ground-truth distribution of visibility.

**Figure 8:**
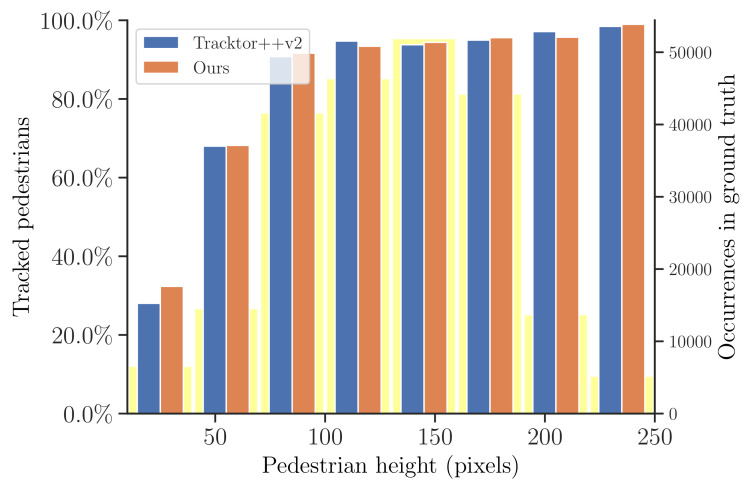
Illustration of the ratio of tracked pedestrians with respect to pedestrian size on the MOT20 training set. The transparent yellow bars indicate the ground-truth distribution of sizes.

At this part, we make a deep analysis of the proposed method in the crowded scenes. We compare our method with the previous work, Tracktor++v2 [11], on the very crowded MOT20 to validate the superiority of our method in tracking the partially occluded pedestrians and the small-looking ones. Since the ground-truth data of the MOTChallenge test sets are not publicly available, we conduct our analysis on the MOT20 training set. We explicitly analyze two difficulties for tracking in crowded scenes, namely tracking partially visible pedestrians and the ones looking small. Figure 7 and Figure 8 report the ratio of tracked pedestrians with respect to pedestrian visibility and size, respectively. For simplicity, we define **pedestrian visibility** as the ratio between non-occluded area and total area of a pedestrian. As shown in Figure 7, when pedestrian visibility >50%, both trackers perform well. As the pedestrian visibility decreases, the advantages of our proposed method gradually emerge. Obvious objects are detected and tracked at the first association, while hard objects (small or partially occluded) are successfully detected and tracked with the help of history-aware masks at the second association. Therefore, our method significantly outperforms Tracktor when the pedestrian visibility is below 0.3. For the **pedestrian size**, we consider the scale of a pedestrian is proportional to its height; therefore, we report the tracked pedestrians percentage with respect to pedestrian height. Here, we only consider objects whose visibility is larger than 0.9. As shown in Figure 8, our method yields better performance. This proves that our method is more advantageous in detecting and tracking small objects.

## 5. Conclusions

In this paper, we propose a simple yet effective method for improving the performance of multiple pedestrian tracking in crowded scenes. The core of our method is the hierarchical association strategy, where the salient objects are directly matched with active trajectories at the first association; the occluded and small ones are progressively identified with the help of spatial cues offered by a history-aware mask at the second association. Moreover, we demonstrate the superior performance of our method in challenging scenarios. We expect our work to inspire future work to pay more attention to the crowded, challenging scenes.

## Figures and Tables

**Table 1:** Results on the MOT16 test datasets. The best and second best results are indicated by bold and underlined numbers, respectively. The arrow ↑ indicates higher values are favored. The arrow ↓ implies low optimal metric values.

Method	Type	MOTA ↑	IDF1 ↑	FP ↓	FN ↓	IDs ↓
FWT [47]	offline	47.8	44.3	8886	85,487	852
GCRA [27]	offline	48.2	48.6	5104	88,586	821
LMP [7]	offline	48.8	51.3	6654	86,245	481
HCC [48]	offline	49.3	50.7	5333	86,795	391
CRFTrack [17]	offline	50.3	54.4	7148	82,746	702
TPM [18]	offline	51.3	47.9	2701	85,504	420
MPNTrack [49]	offline	58.6	61.7	4949	70,252	354
LPC_MOT [50]	offline	58.8	67.6	6167	68,432	435
JCSTD [51]	online	47.4	41.1	8076	86,638	1266
MOTDT [7]	online	47.6	50.9	9253	85,431	792
KCF16 [52]	online	48.8	47.2	5875	86,567	906
PV [53]	online	50.4	47.5	2600	86,780	702
Tracktor [11]	online	54.4	52.5	3280	79,149	682
TrctrD16 [54]	online	54.8	53.4	2955	78,765	645
Tracktor++v2 [11]	online	56.2	54.9	**2394**	76,844	617
GSM [29]	online	57.0	58.2	4332	73,573	**475**
TADAM [33]	online	59.1	**59.5**	2540	71,542	529
**Ours**	online	**59.7**	53.3	3437	**69,227**	885

**Table 2:** Results on the MOT17 test datasets. The best and second best results are indicated by bold and underlined numbers, respectively. The arrow ↑ indicates higher values are favored. The arrow ↓ implies low optimal metric values.

Method	Type	MOTA ↑	IDF1 ↑	FP ↓	FN ↓	IDs ↓
jCC [55]	offline	51.2	54.5	25,937	247,822	1802
FWT [47]	offline	51.3	47.6	24,101	247,921	2648
eTC17 [12]	offline	51.9	50.8	31,572	232,659	3050
JBNOT [16]	offline	52.6	50.8	31,572	232,659	3050
CRF_TRA [17]	offline	53.1	53.7	27,194	234,991	2518
TPM [18]	offline	54.2	52.6	13,739	242,730	1824
MPNTrack [49]	offline	58.8	61.7	17,413	213,594	1185
LPC_MOT [50]	offline	59.0	66.8	23,102	206,948	1122
DASOT17 [56]	online	49.5	51.8	33,640	247,370	4142
MTDF17 [20]	online	49.6	45.2	37,124	241,768	5567
YOONKJ17 [21]	online	51.4	54.0	29,051	243,202	2118
MOTDT17 [7]	online	50.9	52.7	24,069	250,768	2474
FAMnet [22]	online	52.0	48.7	14,138	253,616	3072
Tracktor [11]	online	53.5	52.3	12,201	248,047	2072
Tracktor++v2 [11]	online	56.3	55.1	**8866**	235,449	1987
GSM [29]	online	56.4	57.8	14,379	230,174	**1485**
TADAM [33]	online	59.7	**58.7**	9676	216,029	1930
**Ours**	online	**60.6**	54.3	10,494	**208,861**	2956

**Table 3:** Results on the MOT20 test datasets. Note that the methods marked by * are submitted on CVPR2019 Challenge in which the video sequences are similar to MOT20 with very minor correction. The best and second best results are indicated by bold and underlined numbers, respectively. The arrow ↑ indicates higher values are favored. The arrow ↓ implies low optimal metric values.

Method	Type	MOTA ↑	IDF1 ↑	FP ↓	FN ↓	IDs ↓
IOU_19 * [57]	offline	35.8	25.7	24,427	319,696	15,676
V_IOU * [58]	offline	46.7	46.0	33,776	261,964	2589
MPNTrack [49]	offline	57.6	59.1	16,953	201,384	1210
LPC_MOT [50]	offline	56.3	62.5	11,726	213,056	1562
SORT20 [1]	online	42.7	45.1	27,521	264,694	4470
DD_TAMA19 * [59]	online	47.6	48.7	38,194	252,934	2437
MLT [60]	online	48.9	54.6	45,660	246,803	2187
Tracktor * [11]	online	51.3	47.6	16,263	253,680	2584
Tracktor++v2 [11]	online	52.6	52.7	**6930**	236,680	**1648**
TADAM [33]	online	56.6	51.6	39,407	**182,520**	2690
**Ours**	online	**59.9**	**55.3**	12,458	192,846	2353

**Table 4:** We investigate how spatial–temporal cues improve tracking performances and show the superior performance of our hierarchical association strategy; *w/o His* denotes the proposed model exploits the blank history-aware mask in both associations. **Best in bold**.

Method	MOTA ↑	IDF1 ↑	FP ↓	FN ↓	IDs ↓
Tracktor [11]	70.6	**65.4**	3652	175,955	**1441**
*w*/*o His*	71.2	64.9	**2906**	172,471	1703
Full model	**72.5**	65.0	3736	**163,418**	2062

**Table 5:** Ablation studies on motion models on the MOT17 validation set. The best result in each metric is marked in **bold**.

Method	MOTA ↑	IDF1 ↑	FP ↓	FN ↓	IDs ↓
*w*/*o* CMC & LMM	58.0	51.7	**11,223**	48,090	2658
*w*/*o* LMM	61.4	59.5	12,309	47,349	2682
*w*/*o* CMC	59.6	54.3	11,259	48,066	**2514**
Full model	**62.3**	**63.0**	12,180	**47,349**	2682

## Data Availability

The data presented in this study are openly available in an open access repository at [14,15].
